# Chronic Obstructive Pulmonary Disease and Arthritis Among US Adults, 2016

**DOI:** 10.5888/pcd16.190035

**Published:** 2019-07-18

**Authors:** Yong Liu, Anne G. Wheaton, Louise B. Murphy, Fang Xu, Janet B. Croft, Kurt J. Greenlund

**Affiliations:** 1 Division of Population Health, Centers for Disease Control and Prevention, Atlanta, Georgia

## Abstract

**Introduction:**

More than 54 million US adults have arthritis, and more than 15 million US adults have chronic obstructive pulmonary disease **(**COPD). Arthritis and COPD share many risk factors, such as tobacco use, asthma history, and age. The objective of this study was to assess the relationship between self-reported physician-diagnosed COPD and arthritis in the US adult population.

**Methods:**

We analyzed data from 408,774 respondents aged 18 or older in the 2016 Behavioral Risk Factor Surveillance System to assess the association between self-reported physician-diagnosed COPD and arthritis in the US adult population by using multivariable logistic regression analyses.

**Results:**

Overall crude prevalence was 6.4% for COPD and 25.2% for arthritis. The prevalence of age-adjusted COPD was higher among respondents with arthritis than among respondents without arthritis (13.7% vs 3.8%, *P* < .001). The association remained significant among most subgroups (*P* < .001) particularly among adults aged 18 to 44 (11.5% vs 2.0%) and never smokers (7.6% vs 1.7%). In multivariable logistic regression analyses, arthritis status was significantly associated with COPD status after controlling for sociodemographic characteristics, risk behaviors, and health-related quality of life measures (adjusted prevalence ratio = 1.5, 95% confidence interval, 1.4–1.5, *P* < .001).

**Conclusion:**

Our results confirmed that arthritis is associated with a higher prevalence of COPD in the US adult population. Health care providers may assess COPD and arthritis symptoms for earlier detection of each condition and recommend that patients with COPD and/or arthritis participate in pulmonary rehabilitation and self-management education programs such as the Chronic Disease Self-Management Program, the proven benefits of which include increased aerobic activity and reduced shortness of breath, pain, and depression.

**Summary**

**What is already known on this topic?**

More than 54 million US adults report having arthritis, and more than 15 million US adults report having chronic obstructive pulmonary disease.

**What is added by this report?**

Adults with arthritis are more likely than adults without arthritis to have a higher prevalence of COPD (13.7% vs 3.8%). The relationships remained significant among all subgroups of selected characteristics after we controlled for covariates.

**What are the implications for public health practice?**

Assessment of COPD and arthritis symptoms by health care providers may enhance earlier detection of each condition. Evidence-based self-management programs may ameliorate symptoms.

## Introduction

More than 15 million US adults have chronic obstructive pulmonary disease **(**COPD), and more than 54 million US adults have arthritis (1,2). COPD is characterized by progressive, irreversible airflow limitation and was the fourth leading cause of death in the United States in 2016 (3). Arthritis is a common chronic condition and a leading cause of disability (4). Data from cross-sectional studies suggest that adults with COPD are more likely than adults without COPD to have arthritis (5), including osteoarthritis (6) and rheumatoid arthritis (7). Emerging evidence from longitudinal studies also shows a higher risk of developing COPD among adults with arthritis than adults without arthritis at baseline (8–11). COPD and arthritis share many of the same risk factors, such as sex, age, lower socioeconomic status, tobacco use, obesity, and infection (12–14). Most previous studies assessed the COPD–arthritis relationship by controlling for those common risk factors (9–11). However, few studies, including nationally representative studies, examined the relationship among subgroups with or without those risk factors (5,11). Describing these relationships may inform health care providers to assess COPD and arthritis symptoms for earlier detection of these conditions and provide new insights into preventing them.

The objective of this study was to assess the relationship between self-reported physician-diagnosed COPD and arthritis in the US adult population aged 18 or older in the 2016 Behavioral Risk Factor Surveillance System (BRFSS). We examined the relationship among subgroups with and without the common risk factors.

## Methods

The BRFSS is a random-digital–dialed landline and cellular telephone survey that is conducted by state health departments in collaboration with the Centers for Disease Control and Prevention (CDC) in all 50 states, the District of Columbia, and US territories (www.cdc.gov/brfss). The BRFSS collected data on sociodemographic characteristics, health-related behaviors, chronic diseases, and other conditions among the adult population (aged ≥18) in 2016. Disproportionate stratified sampling of respondents defined by race/ethnicity, sex, or age may vary between states. CDC weighted each sample using design weighting and “raking” weighting methods to obtain a study population representative of each state. Trained interviewers administered standardized questionnaires to all adult respondents. All respondents provided an oral consent statement for participating in the survey. The study was approved as exempt research by CDC’s institutional review board.

### Measures

Respondents who answered yes to the question of whether they had ever been told by a doctor, nurse, or other health professional that they had “chronic obstructive pulmonary disease (COPD), emphysema, or chronic bronchitis” or arthritis (“some form of arthritis, rheumatoid arthritis, gout, lupus, or fibromyalgia”) were determined to have those conditions. Asthma status, a covariable in this study, was based on questions about whether respondents had ever been told they had asthma and “still have asthma.” We categorized responses as current asthma, history of asthma only, or no asthma.

Other covariates were age groups (18–44, 45–64, 65–74, and ≥75 y), sex, race/ethnicity (non-Hispanic white, non-Hispanic black, Hispanic, American Indian/Alaska Native, Asian, and non-Hispanic other), education levels (<high school diploma, high school diploma or GED, some college, and college graduate), and employment status (employed, unemployed, retired, unable to work, and other). Sleep duration was defined according to a response to the question, “On average, how many hours of sleep do you get in a 24-hour period?” We defined short sleep duration as fewer than 7 hours of sleep (15). The number of physically unhealthy days was determined according to the question, “Now thinking about your physical health, which includes physical illness and injury, for how many days during the past 30 days was your physical health not good?” The number of mentally unhealthy days was determined according to the question, “Now thinking about your mental health, which includes stress, depression, and problems with emotions, for how many days during the past 30 days was your mental health not good?” Consistent with previous research (16), frequent physical distress was defined as 14 or more physically unhealthy days, and frequent mental distress was defined as 14 or more mentally unhealthy days. Any leisure-time physical activity was defined according to a yes response to a single question, “During the past month, other than your regular job, did you participate in any physical activities or exercises such as running, calisthenics, golf, gardening, or walking for exercise?” Smoking status was defined by 2 questions: “Have you smoked at least 100 cigarettes in your entire life?” and “Do you currently smoke every day, some days, or not at all?” Respondents were current smokers if they reported having smoked at least 100 cigarettes during their lifetime and currently smoked every day or some days. Former smokers were defined as respondents who reported having smoked at least 100 cigarettes during their lifetime but did not currently smoke. Never smokers were defined as respondents who reported not having smoked at least 100 cigarettes during their lifetime. Body mass index (BMI, kg/m^2^) was calculated from self-reported height and weight and categorized as underweight (BMI < 18.5), normal weight (BMI = 18.5–24.9), overweight (BMI = 25.0–29.9), or obese (BMI ≥ 30.0). Excessive alcohol drinkers were defined as respondents who had any alcohol drinks and were younger than 21, women who were pregnant and reported drinking any alcohol in the previous month, or respondents who were binge drinkers (≥5 drinks for men on an occasion and ≥4 drinks for women on an occasion) or heavy drinkers (≥15 drinks per week for men and ≥8 drinks per week for women during the past 30 days) (17).

In 2016, 486,303 respondents aged 18 or older residing in 50 states, Washington, DC, and the US territories participated in the survey. The median response rate was 47.1% (range, 30.7% in Louisiana to 65.0% in Wyoming) (18). After we excluded respondents with missing values (age [n = 6,697], COPD [n = 156], arthritis [n = 99], or risk behaviors and health-related quality of life [n = 70,577]), we analyzed complete data for 408,774 adult respondents.

### Statistical analysis

We calculated the age-adjusted prevalence of COPD and 95% confidence intervals (CIs) by standardizing to the 2000 projected US population in 5 age groups (18–24, 25–34, 35–44, 45–64, and ≥ 65) by arthritis status and selected characteristics (19). We performed multivariable logistic regression analyses to assess the association of COPD with arthritis after controlling for age, sex, race/ethnicity, education levels, employment status, short sleep duration, frequent mental distress, frequent physical distress, smoking status, BMI category, leisure-time physical activity, excessive alcohol drinking, and asthma status. We conducted all analyses in SAS–callable SUDAAN version 11.0.1 (Research Triangle Institute) to account for the complex sampling design. We determined significant differences at *P* < .05.

## Results

Respondents with arthritis were more likely than respondents without arthritis to be women, aged 45 or older, non-Hispanic white, and not a college graduate, to be retired or unable to work, to have frequent mental or physical distress, to be former or current smokers, to have short sleep duration, to be obese, and to have current asthma or a history of COPD. Respondents with arthritis were less likely than respondents without arthritis to report excessive alcohol drinking or leisure-time physical activity (*P* < .001) (Table 1).

**Table 1:** Distribution of Selected Characteristics Among Adults Aged ≥18, by Arthritis Status, Behavioral Risk Factor Surveillance System, 2016

Characteristic	N^a^	Weighted % (95% CI)	
		Has Arthritis (n = 141,752)	Does Not Have Arthritis (n = 267,022)
**Sex**			
Men	184,406	42.8 (42.3–43.4)	53.1 (52.7–53.5)
Women	224,368	57.2 (56.6–57.7)	46.9 (46.5–47.3)
**Age group, y**			
18–44	109,778	15.1 (14.7–15.6)	56.8 (56.4–57.1)
45–64	154,671	43.8 (43.3–44.4)	30.3 (30.0–30.7)
65–74	85,432	23.1 (22.6–23.5)	8.1 (8.0–8.3)
≥75	58,893	18.0 (17.6–18.4)	4.8 (4.6–4.9)
**Race/ethnicity**			
Non-Hispanic white	319,061	74.4 (73.9–75.0)	61.6 (61.2–61.9)
Non-Hispanic black	32,189	10.8 (10.4–11.2)	11.6 (11.4–11.9)
Hispanic	31,705	9.6 (9.2–10.0)	17.9 (17.6–18.3)
American Indian/Alaska Native	5,961	1.1 (1.0–1.2)	0.9 (0.8–0.9)
Asian	8,768	2.1 (1.8–2.5)	6.0 (5.8–6.3)
Non-Hispanic other	11,090	2.0 (1.8–2.1)	2.0 (1.9–2.1)
**Education level**			
<High school diploma	28,645	15.6 (15.1–16.1)	11.6 (11.3–11.9)
High school diploma or GED	113,502	30.2 (29.8–30.7)	27.3 (27.0–27.7)
Some college	112,976	32.6 (32.1–33.2)	31.4 (31.0–31.7)
College graduate	153,132	21.5 (21.2–21.9)	29.7 (29.4–30.0)
**Employment status**			
Employed	204,591	36.5 (36.0–37.1)	65.4 (65.0–65.7)
Unemployed	15,860	4.5 (4.2–4.7)	5.4 (5.2–5.6)
Retired	125,176	36.7 (36.2–37.2)	11.9 (11.7–12.1)
Unable to work	29,143	15.6 (15.2–16.0)	3.6 (3.4–3.7)
Other	32,279	6.7 (6.4–7.0)	13.8 (13.5–14.1)
**Frequent mental distress**			
Yes	43,085	17.6 (17.2–18.1)	9.6 (9.4–9.9)
No	365,689	82.4 (81.9–82.8)	90.4 (90.1–90.6)
**Frequent physical distress**			
Yes	55,210	26.6 (26.1–27.1)	6.9 (6.7–7.1)
No	353,564	73.4 (72.9–73.9)	93.1 (92.9–93.3)
**Leisure-time physical activity in the past 30 days**			
Yes	309,114	66.7 (66.1–67.2)	80.1 (79.8–80.5)
No	99,660	33.3 (32.8–33.9)	19.9 (19.5–20.2)
**Smoking status**			
Current smokers	61,266	19.3 (18.8–19.7)	15.7 (15.4–15.9)
Former smokers	119,165	34.7 (34.2–35.2)	21.4 (21.1–21.7)
Never smoked	228,343	46.0 (45.5–46.6)	62.9 (62.6–63.3)
**Short sleep duration (<7 hours in 24-hour period)**			
Yes	129,697	39.6 (39.1–40.1)	32.9 (32.6–33.3)
No	279,077	60.4 (59.9–60.9)	67.1 (66.7–67.4)
**BMI, kg/m^2^**			
Underweight (<18.5)	6,586	1.4 (1.3–1.6)	2.1 (1.9–2.2)
Normal weight (18.5–24.9)	129,712	24.2 (23.7–24.7)	36.2 (35.8–36.6)
Overweight (25.0–29.9)	147,838	34.1 (33.6–34.7)	35.6 (35.3–36.0)
Obese (≥30.0)	124,638	40.2 (39.7–40.7)	26.1 (25.8–26.4)
**Excessive alcohol drinking**			
Yes	135,738	28.3 (27.8–28.8)	41.0 (40.6–41.4)
No	266,787	71.7 (71.2–72.2)	59.0 (58.6–59.4)
**Asthma status**			
Current asthma	37,667	14.4 (14.0–14.8)	7.0 (6.8–7.2)
History of asthma only	15,995	4.6 (4.4–4.9)	4.5 (4.4–4.7)
No asthma	355,112	81.0 (80.5–81.4)	88.5 (88.2–88.7)
**Has COPD**			
Yes	33,421	14.8 (14.4–15.2)	3.5 (3.4–3.7)
No	375,353	85.2 (84.8–85.6)	96.5 (96.3–96.6)

Abbreviations: BMI, body mass index; CI, confidence interval; COPD, chronic obstructive pulmonary disease.

a The final unweighted sample size (n = 408,774) was obtained from 486,303 interviewed adults aged ≥18 year and excluded respondents with a missing value for age, COPD, arthritis, risk behaviors, or health-related quality of life. Categories might not sum to the sample total because of missing responses.

**Figure 1:**
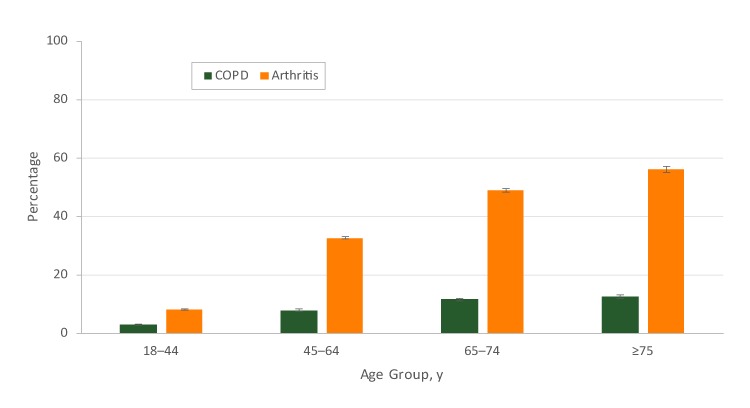
Age-specific percentage of self-reported chronic obstructive pulmonary disease (COPD) and arthritis among US adults aged ≥18, 2016 Behavioral Risk Factor Surveillance System. Error bars indicate standard errors.

The crude prevalence of COPD and arthritis was 6.4% and 25.2%, respectively, among US adults in 2016. Respondents with arthritis had a higher age-adjusted COPD prevalence than respondents without arthritis overall (13.7% vs 3.8%, *P* < .001) (Table 2). In subgroup analyses, we observed a higher prevalence of COPD and arthritis with increasing age (Figure 1).

**Table 2:** Age-Adjusted Prevalence and Adjusted Prevalence Ratio of COPD Among Adults Aged ≥18, by Arthritis Status and Selected Characteristics, Behavioral Risk Factor Surveillance System, 2016^a^

Characteristic	Has Arthritis, % (95% CI)^b^	Does Not Have Arthritis, % (95% CI)^b^	Adjusted Prevalence Ratio (95% CI)^c^
**Total**	13.7 (13.1–14.4)	3.8 (3.7–3.9)	1.5 (1.4–1.5)
**Sex**			
Men	12.7 (11.8–13.7)	3.7 (3.5–3.9)	1.5 (1.4–1.6)
Women	14.4 (13.7–15.3)	3.9 (3.8–4.1)	1.4 (1.3–1.5)
**Age group, y**			
18–44	11.5 (10.5–12.7)	2.0 (1.9–2.2)	2.2 (1.9–2.5)
45–64	15.6 (15.0–16.2)	4.2 (3.9–4.5)	1.5 (1.4–1.6)
65–74	15.3 (14.6–16.1)	8.1 (7.5–8.6)	1.3 (1.2–1.4)
≥75	15.1 (14.2–16.1)	9.4 (8.7–10.1)	1.2 (1.1–1.3)
**Race/ethnicity**			
Non-Hispanic white	13.5 (12.8–14.2)	4.1 (3.9–4.2)	1.4 (1.3–1.5)
Non-Hispanic black	15.8 (13.7–18.0)	3.9 (3.5–4.3)	1.8 (1.5–2.2)
Hispanic	11.6 (9.8–13.6)	2.5 (2.2–2.8)	2.0 (1.6–2.4)
American Indian/Alaska Native	19.1 (14.9–24.2)	5.7 (4.5–7.1)	1.4 (1.0–1.8)
Asian	—d	1.8 (1.2–2.8)	—d
Non-Hispanic other	19.5 (16.5–22.9)	7.3 (5.4–9.6)	1.1 (0.9–1.5)
**Education level**			
<High school diploma	23.2 (21.2–25.4)	6.3 (5.7–6.8)	1.3 (1.2–1.5)
High school diploma or GED	14.2 (13.3–15.3)	4.7 (4.5–5.0)	1.4 (1.3–1.5)
Some college	13.0 (12.0–14.1)	3.8 (3.6–4.1)	1.6 (1.4–1.7)
College graduate	6.6 (5.8–7.5)	1.9 (1.8–2.1)	1.6 (1.4–1.8)
**Employment status**			
Employed	8.6 (7.9–9.3)	2.6 (2.5–2.8)	1.8 (1.7–2.0)
Unemployed	17.5 (15.2–20.1)	5.3 (4.6–6.1)	1.6 (1.3–2.0)
Retired	11.0 (7.8–15.2)	4.8 (3.4–6.8)	1.3 (1.2–1.4)
Unable to work	28.0 (25.2–30.9)	12.6 (11.4–13.8)	1.3 (1.1–1.4)
Other	11.4 (9.8–13.3)	3.3 (2.8–4.0)	1.6 (1.3–2.1)
**Frequent mental distress**			
Yes	23.2 (21.8–24.6)	9.6 (8.8–10.4)	1.2 (1.0–1.5)
No	10.7 (10.1–11.4)	3.3 (3.2–3.4)	1.5 (1.3–1.6)
**Frequent physical distress**			
Yes	23.6 (22.1–25.1)	11.5 (10.7–12.3)	1.2 (1.1–1.3)
No	9.6 (9.0–10.3)	3.1 (2.9–3.2)	1.6 (1.5–1.7)
**Leisure-time physical activity in the past 30 days**			
Yes	11.2 (10.5–11.9)	3.0 (2.9–3.2)	1.5 (1.4–1.6)
No	18.8 (17.5–20.2)	6.2 (5.9–6.6)	1.3 (1.3–1.4)
**Smoking status**			
Current smokers	25.8 (24.4–27.2)	9.7 (9.2–10.2)	1.4 (1.3–1.6)
Former smokers	14.3 (12.3–16.5)	5.1 (4.7–5.4)	1.3 (1.2–1.4)
Never smoked	7.6 (6.9–8.3)	1.7 (1.6–1.9)	1.9 (1.7–2.1)
**Short sleep duration (<7 hours in 24-hour period)**			
Yes	17.7 (16.6–18.7)	4.9 (4.7–5.2)	1.5 (1.4–1.6)
No	9.8 (9.2–10.4)	3.2 (3.1–3.4)	1.4 (1.3–1.5)
**BMI, kg/m^2^**			
Underweight (<18.5)	23.7 (19.9–27.9)	8.1 (6.8–9.6)	1.2 (1.0–1.5)
Normal weight (18.5–24.9)	12.9 (11.8–14.0)	3.6 (3.4–3.9)	1.5 (1.3–1.6)
Overweight (25.0–29.9)	11.5 (10.4–12.7)	3.4 (3.2–3.6)	1.5 (1.3–1.6)
Obese (≥30.0)	15.3 (14.3–16.5)	4.8 (4.5–5.1)	1.5 (1.3–1.6)
**Excessive alcohol drinking**			
Yes	11.4 (10.5–12.3)	3.4 (3.2–3.6)	1.4 (1.3–1.5)
No	14.4 (13.7–15.2)	4.0 (3.9–4.2)	1.7 (1.5–1.8)
**Asthma status**			
Current asthma	34.5 (32.8–36.4)	16.2 (15.4–17.2)	1.3 (1.2–1.4)
History of asthma only	18.8 (16.2–21.6)	8.1 (7.1–9.2)	1.5 (1.2–1.8)
No asthma	8.6 (8.0–9.2)	2.7 (2.6–2.8)	1.5 (1.4–1.6)

Abbreviations: BMI, body mass index, CI, confidence interval; COPD, chronic obstructive pulmonary disease.

a The final unweighted sample size (n = 408,774) was obtained from 486,303 interviewed adults aged ≥18 years and excluded respondents with a missing value for age, COPD, arthritis, risk behaviors, or health-related quality of life.

b Age-adjusted percentages and 95% CIs were standardized to the 2000 projected US population except among age groups.

c Adjusted prevalence ratio and 95% CIs were obtained from a multivariable logistic regression model including age, sex, race/ethnicity, education, employment status, risk behaviors (smoking status, excessive alcohol drinking, physical activity, BMI category, sleep duration), and other conditions (frequent mental distress, frequent physical distress, asthma status, and arthritis status).

d Unreliable estimate because relative standard error was >0.3 or n <50.

**Table:** 

Age Group, y	COPD, % (Standard Error)	Arthritis, % (Standard Error)
18–44	2.8 (0.2)	8.2 (0.3)
45–64	7.9 (0.3)	32.7 (0.5)
65–74	11.6 (0.5)	48.8 (0.7)
≥75	12.6 (0.6)	56.0 (0.9)

**Figure 2:**
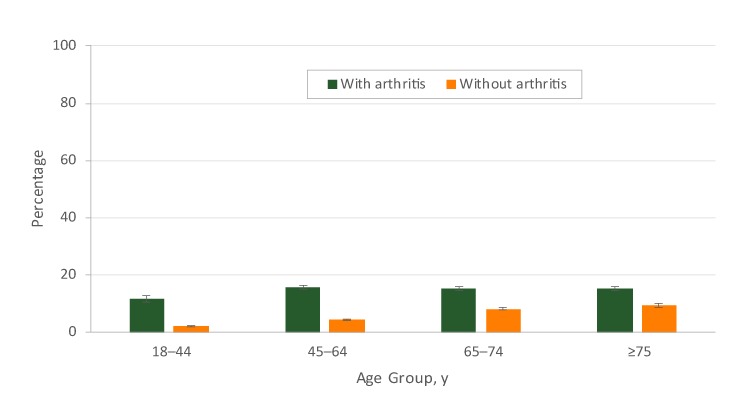
Age-specific prevalence of chronic obstructive pulmonary disease (COPD) among US adults aged ≥18 years, by arthritis status, 2016 Behavioral Risk Factor Surveillance System. Error bars indicate standard errors.

The age-adjusted prevalence of COPD was higher among respondents with arthritis than among respondents without arthritis, regardless of sex, age group, education level, employment status, frequent mental distress, frequent physical distress, leisure-time physical activity, smoking status, short sleep duration, BMI category, excessive alcohol drinking, and asthma status (*P* < .001) (Table 2). The age-adjusted prevalence of COPD was higher among respondents with arthritis than among respondents without arthritis in most racial/ethnic groups (*P* < .001). Although the prevalence of COPD was low in some subgroups (eg, younger adults, college graduates, and respondents who never smoked), respondents with arthritis were significantly more likely than respondents without arthritis to report COPD in these subgroups. For example, among adults aged 18 to 44, the age-adjusted prevalence of COPD was 11.5% for respondents with arthritis and 2.0% for respondents without arthritis (Figure 2).

**Table:** 

Age Group, y	With Arthritis, % (Standard Error)	Without Arthritis, % (Standard Error)
18–44	11.5 (1.0)	2.0 (0.1)
45–64	15.6 (0.6)	4.2 (0.3)
65–74	15.3 (0.7)	8.1 (0.6)
≥75	15.1 (0.8)	9.4 (0.7)

**Figure 3:**
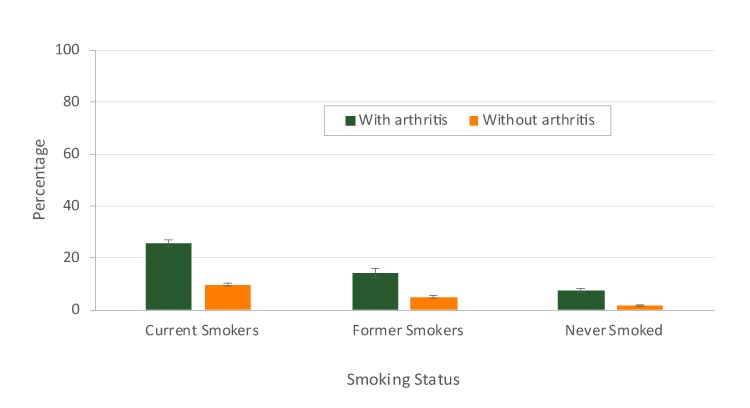
Age-adjusted prevalence of chronic obstructive pulmonary disease (COPD) among US adults aged ≥18 years, by smoking status and arthritis, 2016 Behavioral Risk Factor Surveillance System. Error bars indicate standard errors.

Among never smokers, the prevalence of COPD was 7.6% for respondents with arthritis and 1.7% for respondents without arthritis (Figure 3).

**Table:** 

Smoking Status	With Arthritis, % (Standard Error)	Without Arthritis, % (Standard Error)
Current smokers	25.8 (1.4)	9.7 (0.5)
Former smokers	14.3 (2.0)	5.1 (0.4)
Never smoked	7.6 (0.7)	1.7 (0.1)

In multivariable logistic regression analyses, arthritis was significantly associated with COPD, after controlling for sociodemographics, risk behaviors, frequent mental distress, frequent physical distress, and asthma status (adjusted prevalence ratio = 1.5, 95% confidence interval, 1.4–1.5; *P* < .001) (Table 2). The association remained statistically significant among most subgroups by selected characteristics, including adults aged 18 to 44 and nonsmokers.

## Discussion

Our study demonstrated that the prevalence of COPD was almost 50% higher among adults with arthritis than among adults without arthritis in the United States after adjustment for sociodemographic characteristics, risk behaviors, frequent mental distress, frequent physical distress, and asthma status. Additionally, the relationship between COPD and arthritis was evident in essentially all subgroups of sociodemographic characteristics, risk behaviors, frequent mental distress, frequent physical distress, and a history of asthma. Our results, from a large nationwide sample, are consistent with the results of previous hospitalization and clinical studies of the association between COPD and osteoarthritis or rheumatoid arthritis (5–11). Those previous studies showed only the association between COPD and arthritis in sex and age groups (5,7,8,10,11). Therefore, our results add epidemiological information to the existing literature.

The reason for a positive association between COPD and arthritis is not clear. First, prolonged systemic inflammation may mediate the relationship between arthritis and COPD (8). Second, smoking is a common risk factor for both arthritis and COPD, and it may exacerbate the 2 underlying conditions (20,21). Therefore, smoking cessation is important to preventing and managing arthritis and COPD. Third, stiff joints and joint pain, weak muscles, and poor balance are common symptoms of arthritis. These symptoms can limit a person’s physical activity and further impair respiratory function in people who also have COPD. Conversely, dyspnea or shortness of breath are common symptoms of COPD and can make arthritis worse if patients become more sedentary (22). Therefore, physical inactivity may contribute to the relationship between COPD and arthritis. However, aerobic activity could reduce symptoms of both COPD and arthritis (22,23). Therefore, aerobic physical activity interventions or pulmonary rehabilitation may improve long-term health outcomes for patients with COPD and/or arthritis (22,23). Finally, follow-up care and treatment of one condition may lead health care providers to detect and diagnose comorbid conditions.

Our study showed that the greater prevalence of COPD among adults with arthritis than among adults without arthritis remained significant among all subgroups by selected characteristics in multivariable logistic regression models including sociodemographics, risk behaviors, frequent mental distress, frequent physical distress, and asthma status. It also demonstrated that the relationship between COPD and arthritis was not only among people with common risk factors but also among younger adults and among nonsmokers. Our findings imply that assessment of COPD and arthritis symptoms by a primary care physician might be advised for persons with arthritis or COPD.

Health care providers may also consider referring their patients to evidence-based self-management education programs, such as the Chronic Disease Self-Management Program (CDSMP), which were developed to help people with chronic diseases such as COPD and arthritis manage their symptoms and improve their quality of life (24). A meta-analysis showed that people participating in CDSMP experienced numerous benefits, including reduced shortness of breath, pain, and depression and increased aerobic activity (25).

Our study had several limitations. First, because BRFSS is a cross-sectional survey, we cannot make causal inferences. Second, arthritis was defined as a broad category that included rheumatoid arthritis, gout, lupus, fibromyalgia, or any other form of arthritis. There are more than 100 types of arthritis (26), and our results may not apply to all types. Further research on associations between COPD and various types of arthritis may reveal more insights into the relationship. Third, sample selection bias due to low response rates may have also influenced our results. However, the effect of potential systematic bias was likely limited because the relationship between COPD and arthritis in our study is consistent with the relationship described in previous studies (5,8). Finally, all responses to survey questions in the study were self-reported; as such, they were subject to recall bias, and this bias might have affected our results.

Our results confirmed that arthritis was associated with a higher likelihood of COPD in the US adult population. Health care providers may assess COPD and arthritis symptoms for earlier detection and recommend to their patients with COPD and/or arthritis to participate in evidence-based self-management education programs such as the Chronic Disease Self-Management Program to manage their symptoms, including shortness of breath, pain, and depression, and improve their quality of life.
